# Acute Phase Reactants as Novel Predictors of Cardiovascular Disease

**DOI:** 10.5402/2012/953461

**Published:** 2012-05-06

**Authors:** M. S. Ahmed, A. B. Jadhav, A. Hassan, Qing H. Meng

**Affiliations:** ^1^Department of Medicine, Royal University Hospital, University of Saskatchewan, 107 Wiggins Road, Saskatoon, SK, Canada S7N 5E5; ^2^Department of Pharmacology, University of Saskatchewan, 107 Wiggins Road, Saskatoon, SK, Canada S7N 5E5; ^3^Department of Pathology and Laboratory Medicine, Royal University Hospital, University of Saskatchewan, 103 Hospital Drive, Saskatoon, SK, Canada S7N 0W8

## Abstract

Acute phase reaction is a systemic response which usually follows a physiological condition that takes place in the beginning of an inflammatory process. This physiological change usually lasts 1-2 days. However, the systemic acute phase response usually lasts longer. The aim of this systemic response is to restore homeostasis. These events are accompanied by upregulation of some proteins (positive acute phase reactants) and downregulation of others (negative acute phase reactants) during inflammatory reactions. Cardiovascular diseases are accompanied by the elevation of several positive acute phase reactants such as C-reactive protein (CRP), serum amyloid A (SAA), fibrinogen, white blood cell count, secretory nonpancreatic phospholipase 2-II (sPLA2-II), ferritin, and ceruloplasmin. Cardiovascular disease is also accompanied by the reduction of negative acute phase reactants such as albumin, transferrin, transthyretin, retinol-binding protein, antithrombin, and transcortin. In this paper, we will be discussing the biological activity and diagnostic and prognostic values of acute phase reactants with cardiovascular importance. The potential therapeutic targets of these reactants will be also discussed.

## 1. Introduction

 Since 1930, investigators began to investigate the distant changes occur away from the original site(s) of inflammatory process. The discovery of C-reactive protein (CRP) by Tillett and Francis [1] opened this door wide. These distant systemic changes have been referred to as acute phase response regardless whether they follow an acute or a chronic inflammatory process. Acute phase responses are divided according to the concentrations of many plasma proteins known as acute phase proteins. Acute phase proteins can be defined as those whose plasma protein concentration increase with inflammatory reaction (positive acute phase proteins). However, negative acute phase proteins are those whose plasma protein concentrations decrease with inflammatory reaction. Interleukin- (IL-) 6 is the major stimulator of the production of most acute-phase proteins. Acute phase proteins predict and/or reflect the intensity of cardiovascular diseases. Cardiovascular diseases are accompanied by the elevation of several positive acute phase reactants such as CRP, serum amyloid A (SAA), fibrinogen, white blood cell count, secretory nonpancreatic phospholipase 2-II (sPLA2-II), ferritin, and ceruloplasmin. Cardiovascular diseases are also accompanied by the reduction of negative acute phase reactants such as albumin, transferrin, transthyretin, retinol-binding protein, antithrombin, and transcortin. The concentration of acute phase reactants in plasma varies according to the severity of the cardiovascular disorder and also due to the differences of pattern of production of the individual protein. This explains why an individual biomarker such as CRP elevates in plasma while another under the same category (such as SAA) does not.

## 2. Positive Acute Phase Reactants

### 2.1. C-Reactive Protein (CRP)

In 1930, Tillett and Francis described an acute phase reactant in the serum of patients with pneumonia that they called CRP because of its precipitation with Pneumococcal C-polysaccharide [1]. The gene for CRP has been localized to chromosome 1 [2] and codes for a mature, 206 amino acid polypeptide [3]. The physiological role for human CRP is unknown [4]. CRP is a member of the pentraxin family of proteins and comprises five identical polypeptide chains in a pentameric structure [5]. CRP has a half-life of approximately 19 hours and its plasma levels are determined predominantly by its rate of synthesis in the liver, although CRP is also produced by other cells such as adipocytes [6].

#### 2.1.1. CRP as an Inflammatory Agent

 CRP is an acute phase protein [7, 8] produced in the liver in response to interleukin- (IL-) 6 which is stimulated, in turn, by tumour necrosis factor-*α* (TNF-*α*) and IL-1 [8, 9].

 Recent studies suggest that CRP plays a pivotal role in many aspects of atherogenesis including LDL uptake by macrophage, release of proinflammatory cytokines, expression of monocyte chemotactic protein-1, intercellular adhesion molecule-1, and vascular cellular adhesion molecule-1 [10–12]. Activation of inflammation and the acute phase reaction appear to play an important role, not only in the pathogenesis of atherosclerosis, but also in the initiation of the acute coronary syndrome (ACS) [13, 14]. Cesari et al. suggested that the inflammatory markers CRP, IL-6, and TNF-*α* are independent predictors of cardiovascular events in older persons [14].

#### 2.1.2. Diagnostic Value

 Although CRP is a nonspecific inflammatory marker which may not be a diagnostic marker for cardiac injury but it is a strong independent predictor for cardiovascular risk and events. Epidemiological studies and clinical trials have found that CRP is a strong independent predictor of future cardiovascular risk [15]. Several large epidemiological studies have suggested that CRP measurement predicts the risk of future CV events [16–19] although other investigators failed to identify CRP as a significant independent risk factor [20, 21]. CRP is also an early ischemic marker and elevated CRP is predictive of future adverse events [22, 23]. High-sensitivity CRP (hs-CRP) rises acutely after tissue injury, including myocardial infarction (MI). Intense cytokine production and inflammatory cell infiltration occur in the area of ischemia and necrosis. This increase of hs-CRP levels, in part, correlates with infarct size [24, 25] and with a higher risk of cardiac rupture [26, 27]. In short-term studies of patients with ACS, high CRP concentrations have been shown to be predictive of death, but not recurrent AMI [28, 29]. There are few studies assessing the long-term risks of an elevated CRP in the ACS population [30–34]. CRP levels are influenced by additional factors such as aging, gender, ethnicity, obesity, diabetes, estrogen use, smoking, hypertension, and autoimmune diseases [8]. Serum levels of CRP can increase 1000-fold in febrile illnesses, various inflammatory states, and trauma [35]. CRP can be also used for patients screening in the primary prevention population [36]. Ockene et al. indicated that CRP is generally expressed at low levels (<1 mg/L) in healthy adults and levels remain relatively stable in the absence of an acute inflammatory stimulus [37]. Patients with unstable angina and CRP >3 mg/L at discharge are more likely to be readmitted for recurrent cardiovascular instability or MI within 1 year [38]. Under acute conditions, concentrations of C-reactive protein increased during the first 6 to 8 hours and can reach peak levels approaching 300 mg/L after approximately 48 hours [39].

Pietilä et al. indicated that hs-CRP measurement is the strongest correlative factor for future clinical events due to arterial inflammation, myocardial infarction, unstable angina, stroke, and peripheral vascular disease in both diseased and apparently healthy asymptomatic patients [40]. The CRP plasma level also is the best risk assessment in patients with either stable or unstable angina, long term after myocardial infarction, and in patients undergoing revascularization therapies [41]. One study showed the only independent cardiovascular risk indicators using multivariate, age adjusted and traditional risk analysis were CRP and Total/HDL cholesterol ratio. If CRP, IL-6, and ICAM-1 levels are added to lipid levels, risk assessment can be improved over lipids alone. Moreover, serum CRP may indicate the vulnerability of the plaque [40].

#### 2.1.3. Prognostic Value

 A growing body of studies suggest that elevation of hs-CRP levels predicts a poor cardiovascular prognosis [42]. The extent of the inflammatory response to injury appears to have prognostic significance, which is independent of the extent of myocardial injury. hs-CRP response after MI has been shown to predict future CHD morbidity and mortality independent of infarct size [43].

 CRP is also a predictor of incident type 2 diabetes. As well, it adds a prognostic information on vascular risk at all levels of the metabolic syndrome [44].

#### 2.1.4. CRP Measurement

 As a protein, CRP is quite stable when stored at −70°C. It was shown that reference intervals for hs-CRP determined by using fresh serum samples were identical to those obtained by using samples, from a similar population, that were stored for 4.5 yr [45].

 When CRP was used primarily for measuring states of extremely active inflammation, such as sepsis or arthritis, values of 50–100 mg/L are most relevant [46]. Until the late 1970s, CRP was measured using qualitative or semiquantitative laboratory technique, most commonly latex agglutination, which precluded its use as differential diagnostic test because any degree of inflammation produced positive results [47].

 Although most of the currently CRP is measured by high sensitive methodologies (hs-CRP) such as immunoturbidimetry and immunonephelometry, preanalytic and analytic variations still exist [48]. The detection range of these assays spans from 3 to well over 200 mg/L [8]. These methods still do not have the sensitivity needed for the use of CRP in the assessment of future coronary risk [8]. The most recent hs-CRP assays utilize either antibodies that are labelled with an enzyme (ELISA) or a fluorescent compound or polystyrene beads-coated antibodies to achieve the desired sensitivity [8].

 Continuation in improvement of its sensitivity, precision, accuracy, and standardization is critical. The interpretation, defining cut-off values, and integration into screening panel are essentials in guidance of clinical practice.

The American Heart Association and the Centers for Disease Control and Prevention (AHA/CDC) issued guidelines for the utility of this marker in the primary prevention setting and in patients with ACS [43]. 

The guideline recommended from the laboratory aspects the cut-off values for risk assessment: hs-CRP concentration <1 mg/L are considered low risk, 1–3 mg/L intermediate risk, and >3 mg/L are in high risk.

### 2.2. Serum Amyloid A (SAA)

#### 2.2.1. Biological Activity

 SAA is an acute phase reactant protein and is secreted by macrophages, vascular smooth muscle cells, and endothelial cells [49, 50]. SAA is also expressed in adipose tissue and there is an emerging body of research investigating its role in adipose inflammation and insulin resistance and diabetes [51]. Thus, the association of SAA with CVDs may be either direct from effects of SAA on atherosclerosis, or indirect via effects of SAA on clinical conditions known to confer increased risk for CVDs [51]. SAA is synthesized primarily in liver in response to stimulation by cytokines such as TNF-a, IL-1, and IL-6 [51]. The SAA family comprises acute-phase isoforms (SAA-1 and SAA-2) and a constitutive isoform (SAA-4) [51]. SAA-3 is an isoform that shares approximately 60% homology with the acute-phase isoforms but it is not expressed in humans and it does not appear to be a component of the acute-phase response [51].

#### 2.2.2. SAA as a Proatherogenic Agent

 SAA promotes the chemotaxis for monocytes and neutrophils, stimulation of the production of other proinflammatory cytokines such as IL-1b and TNF-a, and induction of the matrix metalloproteinases (MMPs) [52, 53]. It was also confirmed that SAA promotes thrombosis by increasing tissue factor [54, 55]. SAA alters HDL function by impairing reverse cholesterol transport (RCT) [51]. RCT is well known to be promoted by HDL which plays a pivotal role in lipid metabolism [51]. It was determined that SAA could potentiate atherogenesis at a number of stages. (1) SAA is chemotactic for neutrophils and monocytes. (2) SAA-carrying LDL may have greater proteoglycan-binding affinity leading to increased retention. (3) SAA stimulates the synthesis of biglycan and increases its binding affinity for LDL. (4) SAA can stimulate the production of other proinflammatory cytokines, exacerbating the vascular inflammation. (5) SAA can induce matrix metalloproteinases (MMPs) which can lead to the destabilization of the developing atherosclerotic plaque [51].

#### 2.2.3. Diagnostic Value

 SAA along with CRP are used clinically as inflammatory markers [49]. These proteins usually respond in parallel to a given stimulus; however, the magnitude of the SAA response has been found to be greater than that of CRP [56–58]. Some studies have shown that SAA levels increase with higher degrees of inflammation, even in some noncardiovascular inflammatory conditions, whereas CRP levels remain normal [59, 60]. Thus, SAA is considered more sensitive and useful than CRP as a marker of acute inflammatory response [59, 60]. Casl et al. have conducted a study on early recognition of acute kidney allograft rejection. Their results showed that acute renal allograft rejection induces a dramatic acute phase response [61]. Peak levels of SAA were increased up to 1000-fold above the normal and those of CRP about 100-fold of the normal [61]. An excellent correlation between kidney allograft rejection and SAA reaction was found in this study and monitoring of SAA concentrations in patients with kidney allograft was recommended as a valuable aid in the early diagnosis and prediction of acute allograft rejection [61]. Kosuge et al. indicated that patients with elevated SAA levels had higher rates of adverse events (death, myocardial infarction, or urgent target-vessel revascularization) at 30 days, irrespective of whether CRP was elevated [49]. In contrast, elevated CRP levels with normal SAA were not associated with adverse outcomes at 30 days [49]. The overall conclusion is that SAA is more sensitive than CRP in responding to cardiovascular and noncardiovascular events.

#### 2.2.4. Prognostic Value

 A study has been conducted by Katayama et al. demonstrated a significant association between the SAA level in the acute phase of AMI and clinical prognosis [62]. It also indicated that SAA can predict cardiac death [62]. This study also confirmed a significant positive correlation between the levels of SAA in acute phase AMI and the levels of hs-CRP and peak CK [62]. Liuzzo et al. have also demonstrated that elevation of CRP and SAA at the time of hospital admission predicts a poor outcome in patients with unstable angina [63].

#### 2.2.5. Measurement of SAA

 Kosuge et al. have described the measurement of SAA by latex-enhanced nephelometric immunoassay on a Hitachi 7600 autoanalyzer (Hitachi, Tokyo, Japan) [49]. Casl et al. have established a quantitative determination of SAA by micro-ELISA method with sensitivity of 0.1 mg/1 and precision expressed with CVs between 1.6 and 4.2% [61].

### 2.3. Fibrinogen

 Fibrinogen is a positive acute phase reactant. The association between the high concentration of fibrinogen and risk of cardiovascular disease is well established. The relation was first reported in preliminary results from the Northwick Park Heart Study in 1980 [64].

#### 2.3.1. Biochemistry and Biological Activity

 Fibrinogen is a symmetrical glycoprotein composed of six polypeptide chains of three types: two A*α*, two B*β*, and two *γ* [65]. These chains are held together by disulphide bridges [66]. The total molecular weight of high molecular weight (HMW) fibrinogen is 340,000 daltons [65].

 There are several mechanisms by which fibrinogen may increase cardiovascular risk. First, it binds specifically to activated platelets via glycoprotein IIb/IIIa, contributing to platelet aggregation. Second, increased fibrinogen levels promote fibrin formation. Third, it is a major contributor to plasma viscosity. Finally, it is an acute-phase reactant that is increased in inflammatory states [67].

#### 2.3.2. Diagnostic Value

 The role of plasma fibrinogen as an independent cardiovascular risk factor has been documented [68]. Occlusive thrombi are found in most cases of acute myocardial infarction (MI), sudden cardiac ischemic death, and unstable angina pectoris [69, 70]. Thrombosis is recognized as the central mechanism of these atherosclerotic complications [68, 69].

 Plasma fibrinogen is also associated with traditional risk factors such as smoking, obesity [67]. Fogari et al. found that fibrinogen levels increased with the number of cigarettes smoked. Fibrinogen levels also quickly fall after smoking cessation, suggesting that this rapid fall in level may be a mechanism for the reduction in cardiovascular risk after smoking cessation [71].

 Association between the elevation in fibrinogen level and obesity increases the risk of cardiovascular disease [67]. A reduction in body mass index after a low-calorie diet for 6 months has been accompanied by a fall in fibrinogen level [72]. The Prospective Cardiovascular Munster (PROCAM) study found that individuals who had LDL and fibrinogen levels in the highest tertile had a 6.1-fold increase in coronary risk compared with those in the lowest tertile [73]. The event rate was significantly lower when fibrinogen levels were in the lowest tertile even though LDL remained in the highest tertile [67].

 Association between diabetes and increased plasma fibrinogen levels was found to cause platelet hyperreactivity as fibrinogen is acting as a cross bridge between platelets [67]. This might explain why poor diabetic control is particularly associated with higher levels of fibrinogen [74].

 Gil et al. have determined that increased plasma levels of fibrinogen as an acute phase reactant marker and associated with unfavorable outcome of acute coronary syndrome (ACS) [75].

#### 2.3.3. Prognostic Value and Measurement

 Shi et al. have demonstrated that elevated level of plasma fibrinogen is associated with a worse long-term prognosis in patients who have ACS [76].

 There are four main methods used to measure fibrinogen concentration: clotting rate assays; clottable protein assays; heat-/salt-precipitation and immunological assays. The results obtained can be affected by the assays used, the fibrinogen heterogeneities described above, and other plasma constituents [65].

### 2.4. White Blood Cell (WBC) Count

 It was well established that elevated WBC count is associated with systemic bacterial infections and during inflammatory processes.

#### 2.4.1. Relationship between Total Leukocyte Count and Cardiovascular Disease and Associated Risk Factors

 Rana et al. indicated that coronary heart disease (CHD) is associated with elevated total leukocyte count [77]. The association of leukocyte count with CHD was first reported in the 1920s [78]. A clear and positive correlation between the leukocyte count and risk of CHD has been established in several prospective studies conducted in CHD-free subjects [79–83]. This correlation appears to persist even after adjustment for other risk factors [84–86]. As smoking is a CHD risk factor, WBC count is higher in smokers compared to non- smokers [87]. Madjid et al. confirmed elevated WBC count as an independent risk factor for CHD [87]. A correlation between baseline leukocyte count and the incidence of MI has been also described [87].

 WBC count has been also counted as a prognostic indicator in subjects with stable CHD after a previous MI within 3 or 6 months [88, 89]. The two conditions were associated with elevated total leukocyte count with an increased risk of re-infarction or death [87]. In patients with acute MI, leukocyte count was significantly high at presentation [87]. In a logistic regression model, leukocytosis was an independent predictor of acute MI [87].

 In untreated patients with hypertension, elevated leukocyte count was associated with subsequent cardiovascular morbidity independent of blood pressure levels, smoking, diabetes, lipid levels, and established markers of target organ damage including electrocardiographic left ventricular hypertrophy and glomerular filtration rate [90]. Several cross-sectional studies have shown a positive relationship between systolic blood pressure (SBP) and inflammatory markers including CRP and WBC count [91]. They demonstrated that increased SBP even within the normotensive range is associated with increased WBC count [91]. They indicated that this association persisted after adjustment for age, gender, smoking status, BMI, physical activity level, cholesterol/HDL-C ratio, and cholesterol-lowering medications [91]. An elevated WBC count may also be a marker for a state characterized by increased catecholamine levels or sympathetic nervous system activity [92] which can raise blood pressure and may eventually result in sustained hypertension [93].

#### 2.4.2. Relationship between Differential Leukocyte Count and Cardiovascular Disease and Associated Risk Factors

 Considering differential leukocyte cell count, a correlation was found between moderately elevated eosinophil count and increased risk of disease, as well as between neutrophil, eosinophil, and monocyte (but not lymphocyte) counts and the incidence of disease [87]. In a case-control study, eosinophil counts were significantly higher in those who had severe vasospastic angina pectoris than in those who had mild vasospastic angina pectoris, stable angina, or no angina [94]. After treatment with antianginal drugs, however, the eosinophil counts decreased to control levels. It was also reported by Biasucci et al. that the number of neutrophils was much higher in the patients with acute MI than in those with UA or stable angina [95]. It was observed by Rana et al. that granulocyte count is consistently associated with increased risk for CHD [77]. Rana et al. also revealed that there is no association of monocyte count or lymphocyte count and risk of CHD and CVD [77]. Granulocytes can release a variety of mediators of tissue injury that cause neutrophil stimulation giving rise to additional products with enhanced endothelial injury [96]. Neutrophils can directly cause damage to coronary vascular endothelium by adherence-dependent mechanisms involving the early adhesion molecule *P*-selectin [97]. Granulocytes also release highly cytotoxic-free oxygen radicals and proteolytic enzymes [98]. ROS may influence vascular tone either indirectly by inactivating endothelium-derived relaxing factor [99] and reducing the release of prostacyclin [100] or directly by promoting smooth muscle cell contraction [101]. This may result in the loss of vasodilator, antithrombotic, and antiatherogenic properties of the vascular endothelium [102]. The overall effect is elevating systolic blood pressure even within normotensive range. Intense neutrophil activation in unstable angina or AMI, as manifested by morphologic changes in neutrophils and elastase release, may relate to ongoing *in vivo* cellular activation [103]. Another study has demonstrated that increased neutrophil platelet adhesion may contribute to neutrophil activation in unstable angina [104]. On the other hand, Schillaci et al. suggested a role for monocytes in atherogenesis and vascular thrombosis (through an interaction with platelets) by giving rise to foamy macrophages and ROS [90]. One retrospective study of patients with coronary artery disease (CAD) showed that five-year survival was significantly better for patients who had a normal as compared with a low relative lymphocyte count (92% versus 83%) [105].

### 2.5. Secretory Nonpancreatic Type II Phospholipase A2 (sPLA2-II)

#### 2.5.1. Chemical and Biological Properties

 sPLA2-II is a positive acute phase reactant with properties to catalyze the production of lipid mediators leading to impaired vasodilator function in patients with documented CAD [106].

 PLA2 are ubiquitous enzymes that hydrolyse the *sn*-2-acyl bond of cell membrane phospholipids and lipoproteins and yield free fatty acids and lysophospholipids, precursors of various proinflammatory lipid mediators, including leukotrienes, prostaglandins, and platelet-activating factor [106, 107]. PLA2 exist in many isoforms including the 14 KDa secretory phospholipase A II (sPLA2) and the 85 KDa cytosolic phospholipase A II (cPLA2) [108]. sPLA2-I is described as pancreatic PLA2 since it is expressed in high amounts in pancreas [108]. Proinflammatory cytokines lead to an increase in expression and secretion of sPLA2-II from different organs and tissues, including atherosclerotic plaques [109] where sPLA2 was found to be highly expressed [109, 110]. Extracellular sPLA2-II is mainly localized at sites where it hydrolyses phospholipids from lipoproteins and lipid aggregates retained in the extracellular matrix of the arterial wall. This may be a potential mechanism for *in situ *release of proinflammatory lipids, free fatty acids and lysophosphatidylcholine in regions of apo-lipoprotein B accumulation, which are abundant in atherosclerotic lesions [111]. Additionally it was documented that sPLA2 induces the expression of chemokines and adhesion molecules in microvascular endothelium [112].

#### 2.5.2. Diagnostic Value

Kugiyama et al. have demonstrated that elevated serum level of sPLA2-II is an independent risk factor for CAD [113]. They also indicated that the increase in sPLA2-II serum levels is a predictor of developing clinical coronary events in patients with CAD [113]. Fichtlscherer et al. have referred this association to the fact that elevated serum sPLA2-II causes impairment in systemic endothelial vasodilator function [106]. They also confirmed that sPLA2-II and CRP are sharing this function in terms of being independent predictors of vascular response to acetylcholine [106]. They concluded that both sPLA2-II and CRP can serve as mediators of endothelial dysfunction [106, 114]. The correlation with CRP is much stronger than that with WBC count [115].

 In a large cohort study over 4 years on patients with CHD, a single measurement of sPLA2-II mass and activity at baseline was associated with recurrent events even after controlling other risk factors that might increase risk for CVD. sPLA2-II mass and activity were strongly correlated but the association of mass with secondary CVD event was even stronger [114].

#### 2.5.3. sPLA2-II as a Therapeutic Target

 LY315920 ([[3- (aminooxoacetyl)-2-ethyl-1-(phenylmethyl)-1H-indol-4-yl] oxy] acetate), a pharmacological drug that functions as a selective stoichiometric inhibitor of the catalytic activity of sPLA2-II [108]. LY315920 has been administered orally and intravenously in transgenic mice expressing human sPLA2-II [108]. LY315920 has inhibited the sPLA2-II serum activity in these mice [108]. Some other drugs have several biological and pharmacological properties including the inhibition of sPLA2-II serum activity such as NSAIDs (Alminoprofen (a member of the phenylpropionic acid class of drugs), aspirin [115, 116], heparin [117], and indomethacin [115]). Chemically modified tetracyclines, devoid of antimicrobial properties also inhibit the activity of serum sPLA2-II [118]. Cholesterol lowering agents such as simvastatin and atorvastatin causes a reduction in sPLA2-II [119].

### 2.6. Serum Ferritin

#### 2.6.1. Biological Activity

 Ferritin, which is the major iron storage protein, plays a key role in iron metabolism [120]. Serum ferritin senses the body iron stores and serves as the early sensitive marker for iron deficiency. Serum ferritin is elevated as iron stores rises. Serum ferritin differs markedly from tissue ferritin in molecular weight, iron and carbohydrate content, subunit size, amino acid sequence, and possibly is encoded by genes distinct from those of tissue ferritin [121, 122]. Ferritin maintains up to 4500 atoms of hydrolyzed and polymerized atoms in a soluble form within a protein shell with a hollow interior of approximately 90 $\overset{´}{\text{Å}}$ [123, 124]. Ferritin binds iron in a catalytically inactive manner; accordingly, oxidative reactions cannot be promoted by iron bound to ferritin but by free iron [125]. Tran et al. reported that the synthesis of ferritin is driven by cytokines [126]. Serum ferritin is positively correlated with serum CRP concentrations [121, 127]. Serum ferritin also increases with increasing BMI [128], possibly a result of higher IL-6 levels in heavier subjects. Serum ferritin is inversely proportional to aspirin intake, as persons with higher aspirin use have lower serum ferritin [121, 129] providing the property of aspirin as an anti-inflammatory agent. Many solid conclusions come to the finding that serum ferritin is a positive acute phase reactant and is strongly associated with inflammatory processes including heart diseases and diabetes.

#### 2.6.2. Is There an Association between Serum Ferritin and Cardiovascular Disease?

 The association between serum levels of ferritin and cardiovascular disease is still controversial and requires further investigation. From a study on Finnish subjects (a total of 1931 unselected men) without symptoms of CHD during an average followup for 3 years, It was reported that 2.2 times greater levels of cardiovascular disease were observed in the group with high serum iron (indicative of elevated serum ferritin) compared to the group with low serum iron [130, 131]. Serum ferritin was reported to be associated with cardiovascular disease and cardiovascular mortality [132]. Salonen et al. also reported that increases in serum ferritin accelerate the oxidation of LDL-cholesterol [131]. This oxidized LDL-cholesterol-induced inflammation in blood vessels, including the progression of atherosclerosis. This role is thought to be due to prooxidant properties [130]. In patients with serum ferritin concentrations >200 ng/mL, the risk of myocardial infarction was 2.2 times greater than the patients with serum ferritin levels <200 ng/mL [130]. This indicated that serum ferritin indirectly enhances the role of LDL-cholesterol in the induction of cardiovascular diseases [130]. This role is further enhanced by the elevation of hsCRP accordingly [130].

 In a prospective study performed in a French population, however, Galan et al. failed to find a positive association between serum ferritin and ischemic heart disease [133]. These findings matched well with the suggestions of Sempos et al. [134], 5 years earlier, as the results from the two studies did not support the hypothesis that positive body iron stores, as measured by serum ferritin, are associated with an increased risk of CVD, CHD, or MI death.

 Dominguez-Rodriguez et al. suggested even more extreme finding that major adverse cardiovascular events is associated with lower serum ferritin levels in a study on a total of 196 and 30 days followed-up patients with a first non-ST elevation ACS [135]. Their observation was supported by an *in vitro* study that iron deficiency enhances atheroma inflammation through p38 mitogen activated protein kinase-nuclear factor-*κ*B-extracellular matrix metalloproteinase inducer/matrix metalloproteinase-9 pathway [136].

### 2.7. Serum Haptoglobin

#### 2.7.1. Biological Activity and Structure

 Serum Haptoglobin (Hp) has been identified as a positive acute phase reactant. Hp is an abundant plasma protein which binds with high affinity to hemoglobin [137, 138]. The binding of Hp to hemoglobin can detoxify hemoglobin released into circulation during *in vivo* hemolysis. The binding of Hp to hemoglobin serves to decrease the ability of iron derived from hemoglobin from carrying out oxidative reactions providing that the heme iron in hemoglobin is a very potent oxidant [139]. In other words, Hp function is binding and scavenging of free hemoglobin through the liver or circulating monocytes [140, 141]. In man, two alleles at the Hp genetic locus denoted 1 and 2 exist [137]. The protein product of the Hp 2 allele is defective in its ability to block oxidative reactions mediated by iron-derived hemoglobin [137]. The Hp 1-Hb complex is relatively redox inert, while the Hp 2-Hb complex contains non-transferrin bound redox-active iron [142]. Boretti et al. found that Hp binding to Hb is sufficient to prevent the generation of oxidant species from cell-free Hb that would otherwise mediate hypertension and other adverse vascular outcomes [143]. Interestingly, Boretti et al. also showed that Hp-bound Hb has a very high oxygen affinity and the Hp-Hb complexes stimulate the production of the endogenous antioxidant NO from nitrite [143, 144]. In addition, the heme-binding protein hemopexin has evolved to mop up the toxic porphyrin heme ring released from decomposing cell free Hb, and heme-metabolizing enzymes, such as heme oxygenase-1, provide a functional antioxidant effect that is protective to vascular health [145]. Finally, plasma transferrin protein sequesters and safely transports elemental iron released from the heme ring, one of the most oxidative substances in the human body [145].

#### 2.7.2. Hp-Cardiovascular Disease Association

It was determined in many multiple independent longitudinal studies that the Hp genotype is an independent determinant of the risk of incident cardiovascular disease in individuals with diabetes mellitus (DM) [146–151]. These studies have shown that DM individuals with the Hp 2-2 genotype have a 2–5-fold increased risk of MI, stroke, and cardiovascular death as compared to Hp 1-1 or Hp 2-1 individuals. It was also demonstrated that Hp phenotype is predictive of development of microvascular complications in DM [152]. They found that patients who are homozygous for the Hp 1 allele are at decreased risk for developing retinopathy and nephropathy [152]. This effect, at least for nephropathy, has been observed in both type 1 and type 2 DM [153]. Furthermore, the Hp phenotype may be predictive of development of macrovascular complications in DM [152]. Hp can also bind to HDL (most likely via an interaction with helix 6 of ApoA1) and thereby serve to tether hemoglobin to HDL [137]. In Hp 2-2 DM individuals, there is impairment in the clearance of Hp-hemoglobin due to a decreased uptake of the complex by the CD163 Hp-hemoglobin scavenger receptor present on monocytes and kupfer cells [137]. HDL in these Hp 2-2 DM individuals is dysfunctional in terms of its ability to promote reverse cholesterol transport which is believed to be the primary function of HDL [137]. It was also demonstrated that development of restenosis after percutaneous coronary angioplasty is significantly decreased in DM patients with the 1-1 Hp phenotype [154]. On contrary, Bacquer et al. found that Hp 1-1 individuals are at elevated risk for CHD mortality in a 10-year followup study [155]. They postulated that Hp 2-2 might play a role in early atherogenesis, but Hp 1-1 has a predominant influence in more advanced and life-threatening stages of the disease [155]. They also concluded that Hp 1-1 is of greater prognostic importance for CHD death than classical risk factors, such as smoking, obesity, and diabetes [155].

### 2.8. Ceruloplasmin

#### 2.8.1. Chemical Structure, Function, and Biological Activity

 Ceruplasmin (Cp) is a positive acute phase reactant with bactericidal activity [156]. Cp is a 132-kDa monomer composed entirely of three 42–45-kDa domains with high amino acid sequence homology (about 40%) to each other [156]. Cp is an abundant plasma protein that contains seven copper atoms per molecule and accounts for 95% of the total circulating copper in healthy adults [157, 158]. The physiological function activities of Cp include copper transport, coagulation, angiogenesis, defense against oxidant stress, and iron homeostasis [156]. The “ferroxidase” activity of Cp catalyzes oxidation of Fe^2+^ to Fe^3+^ [157] and is thought to facilitate *in vitro* loading of iron into the iron transport and storage proteins transferrin and ferritin [159].

#### 2.8.2. Serum Cp Predicts Cardiovascular Events

Tang et al. measured serum Cp levels in 3,569 consecutive stable patients undergoing elective coronary angiography with available leukocyte counts and hs-CRP levels [160]. The role of Cp in predicting incident major adverse cardiac events over 3 years of followup was evaluated [160]. A strong positive correlation with hsCRP was observed [160]. Cp level (Quartiles 4 versus 1) was associated with almost 3-fold increase in risk of future major adverse cardiovascular events at 1 year [160]. After adjusting for traditional risk factors including hsCRP, and total leukocyte count, Cp remained independently predictive of major adverse cardiovascular events at 1 year (HR 2.51, 95% CI 1.40–4.49, *P* = 0.001) and at 3 years (HR 1.48, 95% CI 1.01–1.26) [160]. This study concluded that serum Cp independently predicts cardiovascular events.

 Adelstein et al. and Bustamante et al. observed substantial hypercupremia after MI [161, 162]. Since that report, high serum Cp levels were found in patients with multiple cardiovascular disorders including arteriosclerosis [163], abdominal aortic aneurysm [164], unstable angina [165], and vasculitis and peripheral arterial disease [166]. Prospective studies also showed that serum Cp may be an independent risk factor for cardiovascular disease [162]. A prospective Finnish study verified that serum copper concentration was an independent risk factor for ischemic heart disease [167]. The association between Cp and cardiovascular events is well explained by describing a role for Cp in LDL oxidation by vascular cells (free radical-mediated, transition metal-dependent mechanism) [156]. A model for LDL oxidation by Cp in an atherosclerotic lesion was described that shows that Cp is synthesized by macrophages which are activated by TNF-a (proinflammatory cytokine) or by bacterial lipopolysaccharide (cell wall product of infectious organism) [156]. This secreted Cp [168] along with redox-active copper [169], are required for vascular cell-mediated LDL oxidation.

#### 2.8.3. Prognostic Value of Serum Cp

Prognostic value of serum Cp has been compared against that of CRP and fibrinogen in 40 patients of unstable angina over 12 months period [170]. All acute phase proteins were measured in the total study group and in the subgroup with normal troponin T [170]. The levels of serum Cp were found elevated when compared against those in stable angina patients [171]. Serum Cp increased significantly at 72 hours and reach peak levels at 7 days, both in the total study group and in the subgroup with normal troponin T [170]. On the other hand, CRP and fibrinogen serum levels elevated way earlier than those of Cp and last to a way shorter period [170]. All values of acute phase proteins were similar during the in-hospital course [170]. However, CP but not CRP and fibrinogen levels showed a prognostic value because they were significantly higher in patients with complications in both groups, and logistic regression analysis showed that CP levels at 72 hours were the most important factor related to 12-month prognosis [170].

 The role of serum copper in a 2-year progression of atherosclerotic carotid lesions (measured by ultrasonography) in eastern Finnish men was examined by Salonen et al. [172]. An accelerated progression of atherosclerosis was seen only in patients with both high serum LDL and high serum copper [172]. They thought that the prooxidant activity of the copper may be due to a synergism of copper and LDL [173]. Mezzetti et al. also examined the importance of serum CP and LDL oxidation in lesion development in patients undergoing endarterectomy for internal carotid stenosis [174]. They reported that serum CP and LDL lipid peroxide levels measured 24 or 72 hours after surgery were highly correlated with the percentage renarrowing measured after 12 months [174].

 Taking together, these studies suggest that the contribution of CP to the risk of CVD is not independent but rather depends on the lipoprotein profile and possibly other factors [170].

## 3. Negative Acute Phase Reactants

### 3.1. Serum Albumin

 Serum albumin is a negative acute phase protein synthesized in liver. Lower levels of serum albumin within the “normal” range are associated with increased risk of all-cause and cardiovascular mortality [175], as well as with coronary heart disease (CHD) [176, 177] and stroke incidence [178]. These associations persist after adjustment for other known risk factors and preexisting disease and after exclusion of early mortality [175, 177].

#### 3.1.1. Chemistry and Biological Activity

 Serum albumin level accounts for about 50–60% of the serum proteins with a range of 35–50 g/L [179, 180]. Human serum albumin (HSA), a protein of molecular weight 65 KDa, consists of 585 amino acids and one free thiol (Cys 34). The disulphides are positioned in a repeating series of nine loop-link-loop structures centered around eight sequential Cys-Cys pairs to form heart-shaped [179]. Plasma proteins have been suggested as the major extracellular antioxidants in extracellular fluid [179]. Among them, albumin, transferrin, and ceruloplasmin, can sequester transition metals, preventing them from acting as catalysts in highly reactive biochemical reactions [179]. Several studies have suggested that albumin as a powerful oxidants scavenger in human plasma can inhibit hydroxyl radicals, peroxyl radicals, and HOCl [179, 181].

#### 3.1.2. Serum Albumin-Cardiovascular Disease Association

 Substantial evidence support the significant inverse relation between serum albumin level and risk of coronary heart disease as well as cancer, other causes of death, and all-cause mortality [182–184]. Høstmark [185] reported that albumin as an extracellular antioxidant does have a cardioprotective role against lipid peroxidation [185].

 In patients with renal failure, there is a pathophysiological link between CVD, malnutrition, inflammation [186] and serum levels of CRP [187–189]. Additionally, a low level of serum albumin is also a strong predictor of morbidity and mortality in patients with kidney failure [190–192]. Hypoalbuminemia of kidney failure, in part, may be a consequence of activation of the acute phase response and may represent a chronic inflammatory state [193–195]. Kim et al. suggested possible mechanisms that might link hypoalbuminemia with CVD [196].

(1) Cause-Effect Association:

(i) *Albumin, Atherogenic Lipids and Lipoproteins Association.* A significant inverse relationship between serum albumin and lipoprotein (a) has been reported in patients with nephrotic syndrome [197] and in dialysis patients [198, 199]. Stenvinkel et al. showed that an elevated rate of LDL-Apo B production is highly correlated to the prevailing serum albumin levels in patients with nephrotic syndrome [200]. It was shown that by increasing serum albumin levels in continuous ambulatory peritoneal dialysis patients, serum lipoprotein (a) levels were decreased [201]. These findings have been confirmed these findings in humans and rats [202, 203].

(ii) *Albumin-Fibrinogen Association. *Fibrinogen levels correlate inversely with serum albumin levels in patients with nephrotic syndrome [204] and in continuous ambulatory peritoneal dialysis patients [205]. Pickart and Thaler. showed that addition of albumin abolished the free fatty-acid-associated increase in fibrinogen synthesis in mouse liver slices [206]. The intravenous infusion of albumin solution to seriously injured patients caused a significant fall in fibrinogen concentration [207]. Kim et al. suggested that hypoalbuminemia increases plasma fibrinogen levels [208].

(iii) *Albumin and Platelet Aggregation*. There are several reports that platelet aggregation in patients with nephrotic syndrome and in patients on continuous ambulatory peritoneal dialysis, were inversely proportional to the serum albumin concentration [209–211]. Several investigators reported that the addition of albumin *in vivo *[212] or *in vitro *[210, 213, 214] corrected this defect. Schieppati et al. showed that the addition of albumin to platelet-rich plasma from patients with nephrotic syndrome, or the intravenous infusion of albumin in quantities sufficient to correct hypoalbuminemia, also diminished the excessive production of prostaglandin metabolites by nephrotic platelets [215]. Albumin is a lipooxygenase inhibitor in serum [216], which suggests that hypoalbuminemia increases synthesis of leukotrienes [196].

(iv) *Albumin and Blood Viscosity*. Groth demonstrated that infusion of albumin or dextran 40 in 14 patients with various diseases decreased blood and plasma viscosity [217]. Elevated plasma and whole blood viscosity has been reported in patients with nephrotic syndrome [218]. Joles et al. reported that albumin deficiency is accompanied by blood hyperviscosity, possibly by increasing red cell lysophosphatidylcholine [219]. Continuous ambulatory peritoneal dialysis patients had higher whole-blood viscosity than control subjects when all samples were reconstituted to 70% hematocrit [220]. Plasma viscosity was also higher in continuous ambulatory peritoneal dialysis patients than in hemodialysis patients and controls [220]. In the study of Gordge et al., plasma viscosity correlated significantly with the degree of proteinuria in 21 diabetic patients with renal failure [221].

(2) Effect-Effect Association. Serum albumin was negatively correlated with C-reactive protein (CRP) [222, 223]. However, infusion of albumin in patients with hypoalbuminemia did not have an effect on the level of CRP [205]. These findings suggest that the correlation between albumin and CRP may be secondary to inflammation rather than a direct effect of hypoalbuminemia. As well, diabetes and cigarette smoking can cause hypoalbuminemia and atherosclerosis despite the fact that they are not major determinants of hypoalbuminemia in dialysis patients [224].

### 3.2. Transthyretin

#### 3.2.1. Chemistry and Biological Activity

 Transthyretin (TTR) was formerly called prealbumin (since it migrates closer to the anode compared to albumin on serum protein electrophoresis), but this term is misleading as TTR is not a precursor of albumin [225]. TTR is a globular, non-glycosylated protein with a molecular mass of 54.98 kDa [226]. The total mass is approximately 76 kDa with a complexed molecule of retinol-binding protein (RBP; 21 kDa) [227], which is still small enough to diffuse out of the vascular space as readily as albumin (66.3 kDa) or transferrin (79.6 kDa); slightly less than 50% of each of these proteins is normally intravascular as a result [228]. TTR is a negative acute phase protein synthesized primarily in the liver, except for tiny amounts produced by the choroid plexus and the retina [225, 229]. In serum, TTR transports thyroxine and retinol (along with RBP) [230]. The TTR protein circulates as a homotetramer, in which each monomer comprises 127 amino acids arranged as eight antiparallel *β* pleated sheet domains [231]. The TTR gene is located on chromosome 18 and contains four exons [225]. Both wild type and mutated TTR can form amyloid, which suggests that this highly structured protein is innately prone to form *β*-pleated sheet fibrils [232, 233]. Alterations in the primary structure of the TTR protein (owing to TTR mutations) can result in greatly accelerated amyloid formation and are the origin of all symptomatic cases of hereditary transthyretin-related amyloidosis (ATTR) [234] which can consequently cause organ dysfunction [225].

#### 3.2.2. Common Associated-TTR Amyloidoses with Emphasis on CV Manifestations

 TTR is associated with two distinct forms of cardiac amyloidoses: hereditary transthyretin-related amyloidosis (ATTR) which is caused by mutations in the TTR gene, which encodes TTR [225, 235], whereas systemic senile amyloidosis (SSA) is not inherited and is associated with wild type TTR [235]. In patients with ATTR, amyloid can infiltrate any or all of the cardiovascular structures, including the conduction system, the atrial and ventricular myocardium, valvular tissue, and the coronary and large arteries [236, 237]. Branch bundle block leading to atrioventricular and sinoatrial block are the fate of the conduction system compromise [225]. Myocardial infiltration results in a progressive increase in the thickness of the left and right ventricular walls and of the interatrial septum and is associated with worsening hemodynamic impairment [225]. In a study of 38 patients with ATTR-related cardiomyopathy, only one patient (3%) showed a dip plateau in the right ventricular pressure curve profile; 13 patients (34%) showed raised pulmonary capillary wedge pressures, and 14 (47%) showed a Doppler restrictive filling pattern. Interestingly, 11 (29%) of the patients did not display any abnormalities in diastolic function—at least when at rest [238]. LVEF is generally normal or only mildly reduced in patients with ATTR cardiomyopathy [239]. Amyloid infiltration of cardiac valves leads to the formation of nodules or diffuse thickening of the leaflets, accompanied by variable degrees (generally mild) of valvular regurgitation [225]. The clinical spectrum of cardiovascular involvement is wide, ranging from asymptomatic atrioventricular and bundle branch block to severe, rapidly progressive heart failure owing to restrictive cardiomyopathy [225].

#### 3.2.3. Diagnosis

 Diagnosis of systemic amyloidosis is based on monoclonal gammopathy by immunoelectrophoresis, immunofixation on serum and urine, or mutant transthyretin gene as potential risk factors for amyloid disease, and confirmed by positive Congo red staining of any biopsy (periumbilical fat aspiration, rectum, target organ) [240].

#### 3.2.4. Transthyretin as a Therapeutic Target

**Fibril Disrupters**

Recently, 4′-deoxy-4′-iododoxorubicin (IDOX) has been proved useful as a tool for disrupting TTR amyloid fibrils [173]. The amyloid disrupters might also prove useful for amyloidoses associated with other protein precursors, such as the L protein in Alzheimer and the prion scrapie protein [173]. Due to its cardiotoxicity, IDOX analogues able to disrupt amyloid fibrils are needed [173].

**TTR Stabilizers**

Diclofenac is a good example for TTR stabilizers. Diclofenac is an NSAID approved by FDA [241]. TTR-diclofenac complex revealed that the ring with the acetate function is positioned at the innermost part of the binding site, allowing strong hydrogen bond interaction between the drug COOH group and the side chain of Thr119 [241]. In this case the two phenyl rings and the chlorine atoms provide extensive van der Waals interactions with the protein residues that form the P1 and P3 pockets [241]. Diflunisal, which is another nonsteroidal NSAID, has been found to stabilize the tetrameric structure of TTR [242], that is, reduces tetramer dissociation and subsequent monomer misfolding and aggregation into amyloid [236, 242].

#### 3.2.5. Laboratory Assays for TTR

 The methods most commonly used at present are immunonephelometry (IN) and immunoturbidimetry (IT) [243]. Preanalytical factors may also influence plasma concentrations of proteins and other macromolecules [243]. Of particular importance is body position; individuals who have been standing or recumbent for long periods of time have higher or lower concentrations, respectively [243]. It is generally recommended that blood specimens for assay of plasma proteins be drawn after approximately 15–20 min in the sitting position if possible [243].

### 3.3. Serum Transferrin

#### 3.3.1. Chemistry and Biological Activity

 The transferrin family (Trf) constitutes the major iron transport and/or scavenging system in vertebrates and some invertebrates [244]. However, Trf can also function as an iron chelator, which contributes to host defense by limiting iron availability for microbial pathogens [244]. Trf is a negative acute phase reactant. Trf is a soluble glycoprotein and a bilobal molecule, that is, it contains an N-terminal (amino acids 1–336) and a C- terminal (amino acids 337–679) globular domain [245]. Each domain contains a metal-binding site and each lobe binds one iron atom [245]. Amino acid sequences of Trf from several species, including human, bovine, rabbit, and chicken, show a high degree of sequence similarity [246]. Under normal conditions, these two high-affinity-iron binding sites prevent the existence of measurable amounts of unbound iron in the plasma [245]. The main source of iron for Trf is catabolism of nonviable red blood cells and its main destination is the erythroid marrow [245]. Serum Trf transport iron from neutral biological fluids to the cytoplasm by receptor-mediated endocytosis [247]. Free iron is capable of stimulating the production of free radicals which cause oxidative damage such as lipid peroxidation [245].

#### 3.3.2. Transferrin-Cardiovascular Disease Association

 Trf is one of the most important antioxidants and acts by sequestration of iron in a redox-inactive form [248]. Apotransferrin, at physiological concentrations (2-3 mg/mL) is known to inhibit lipid peroxidation in the liposome model by iron-binding [248]. However, at higher concentrations, apotransferrin does not inhibit lipid peroxidation any further, while at low concentrations, apotransferrin reduces antioxidant capacity in a concentration-dependent manner [248]. This finding was clinically relevant in diseases that are associated with lower plasma transferrin concentrations as a result of either a decreased synthesis or increased breakdown such as type 1 and type 2 diabetes mellitus [248]. It has been shown that oxidative damage to transferrin by neutrophil-derived superoxide increases ferrous iron release [249]. *In vitro* glycation of hemoglobin also results in a higher release of iron. Purified hemoglobin from diabetic patients compared to normal individuals contains more free iron, which increases progressively with the extent of the disease [250]. As Trf is a negative acute phase protein, it is downregulated in inflammatory diseases such as diabetes [248]. Moreover, oxidative damage results in protein fragmentation and nephropathy which can lead to loss of transferrin in the urine of type 1 and type 2 diabetic patients [248]. Interestingly, microtransferrinuria seems to be a more sensitive index for renal dysfunction than microalbuminuria [248, 251–255]. The low concentration of transferrin often found in the disease also appears to reflect the increased iron stores, and the concentration returns to normal after iron-depletion therapy. It seems unlikely that hemochromatosis is related to a primary defect in transferrin [256]. Cartei et al. demonstrated that venesection therapy causes the rise of plasma transferrin due to the removal of iron overload and not to venesection *per se* [256]. Another possible defect in hemochromatosis is the decreased ability of reticuloendothelial cells to retain iron [257]. The result of this defect is a return of greater amounts of iron from stores to plasma ferritin, increasing its iron saturation and thus causing more iron saturation and directing more iron into hepatocyte storage [256]. Batey et al. have confirmed the existence of a nontransferrin-bound iron (NTBI) fraction in the sera of patients with untreated or inadequately treated primary hemochromatosis which may, in part explain the findings in primary hemochromatosis [257].

## 4. Conclusion

 The presence and intensity of inflammation of cardiovascular disorders can be reflected by acute phase changes. Acute phase responses have long been used as a clinical guide to diagnosis, management, and prognosis. Not only this, but also they can be used to predict a new-onset cardiovascular disorder.
